# Serum and Cerebrospinal Fluid Biomarkers in Neuromyelitis Optica Spectrum Disorder and Myelin Oligodendrocyte Glycoprotein Associated Disease

**DOI:** 10.3389/fneur.2022.866824

**Published:** 2022-03-23

**Authors:** Alessandro Dinoto, Elia Sechi, Eoin P. Flanagan, Sergio Ferrari, Paolo Solla, Sara Mariotto, John J. Chen

**Affiliations:** ^1^Neurology Unit, Department of Neuroscience, Biomedicine and Movement Sciences, University of Verona, Verona, Italy; ^2^Department of Medical, Surgical and Experimental Sciences, University of Sassari, Sassari, Italy; ^3^Department of Neurology, Mayo Clinic College of Medicine and Science, Rochester, MN, United States; ^4^Department of Laboratory Medicine and Pathology, Mayo Clinic College of Medicine and Science, Rochester, MN, United States; ^5^Departments of Ophthalmology and Neurology, Mayo Clinic College of Medicine and Science, Rochester, MN, United States

**Keywords:** NMOSD, MOGAD, AQP4, biomarkers, neurofilament light chain, glial fibrillary acid protein, cytokines, complement

## Abstract

The term neuromyelitis optica spectrum disorder (NMOSD) describes a group of clinical-MRI syndromes characterized by longitudinally extensive transverse myelitis, optic neuritis, brainstem dysfunction and/or, less commonly, encephalopathy. About 80% of patients harbor antibodies directed against the water channel aquaporin-4 (AQP4-IgG), expressed on astrocytes, which was found to be both a biomarker and a pathogenic cause of NMOSD. More recently, antibodies against myelin oligodendrocyte glycoprotein (MOG-IgG), have been found to be a biomarker of a different entity, termed MOG antibody-associated disease (MOGAD), which has overlapping, but different pathogenesis, clinical features, treatment response, and prognosis when compared to AQP4-IgG-positive NMOSD. Despite important refinements in the accuracy of AQP4-IgG and MOG-IgG testing assays, a small proportion of patients with NMOSD still remain negative for both antibodies and are called “seronegative” NMOSD. Whilst major advances have been made in the diagnosis and treatment of these conditions, biomarkers that could help predict the risk of relapses, disease activity, and prognosis are still lacking. In this context, a number of serum and/or cerebrospinal fluid biomarkers are emerging as potentially useful in clinical practice for diagnostic and treatment purposes. These include antibody titers, cytokine profiles, complement factors, and markers of neuronal (e.g., neurofilament light chain) or astroglial (e.g., glial fibrillary acidic protein) damage. The aim of this review is to summarize current evidence regarding the role of emerging diagnostic and prognostic biomarkers in patients with NMOSD and MOGAD.

## Introduction

The term neuromyelitis optica (NMO) was first used in 1894 by Devic and his fellow, Fernand Gault, to propose a distinct disease entity characterized by simultaneous myelitis and bilateral optic neuritis (1). From Devic's first report until 2004, NMO remained an elusive condition that many thought was a monophasic, more aggressive variant of multiple sclerosis (MS). A major landmark in NMO history was the discovery, by Lennon et al. (2), that sera from patients with NMO outlined microvessels, pia, subpia, and Virchow-Robin spaces when tested on tissue-based indirect immunofluorescence. The putative agent of NMO, aquaporin-4 antibodies (AQP4-IgG), was subsequently found to bind the AQP4 water channel (3). AQP4 is highly expressed in the foot processes of astrocytes, particularly in the domains that interacts with dystrophin-associated proteins and microvessels. The discovery of AQP4-IgG led to the evidence that (1) NMO with positivity for AQP4-IgG is a predominantly an astrocytopathy, and (2) AQP4-IgG is both the pathogenetic cause and the biomarker that defines a distinct disorder which differs from MS.

Whilst NMO was initially defined by the occurrence of longitudinally extensive transverse myelitis (LETM) and optic neuritis, the development of more specific assays, particularly cell-based assays (CBAs) with transfected HEK-293 cells expressing AQP4 (4), led to the realization that the spectrum of disorders associated with AQP4-IgG was broader than previously thought, encompassing limited forms of the disease (e.g., isolated optic neuritis or isolated myelitis), and also brain and brainstem involvement (previously regarded as an exclusion criteria for NMO). These concepts were reflected by the evolution of the diagnostic criteria in 2006 (5) and 2015 (6), the latter emphasizing the importance of AQP4-IgG serostatus, and the adoption of the new nomenclature of “neuromyelitis optica spectrum disorder” (NMOSD) including both AQP4-IgG seropositive and seronegative cases.

About 20% of patients who are diagnosed with NMOSD according to the 2015 NMOSD criteria are seronegative for AQP4-IgG, and among these seronegative patients, about 30% will bear antibodies directed against myelin oligodendrocyte glycoprotein (MOG), which is predominantly expressed in oligodendrocytes or, more rarely, in soluble isoforms (7). The biological role of MOG is still unclear and may represent an adhesion molecule. MOG was initially detected by enzyme-linked immunosorbent assay (ELISA) and immunoblotting, but these assays recognized non-native and non-pathogenic MOG epitopes, probably due to missing glycosylation and incorrect antigen structure. Consequently, MOG antibodies were detected on these older assays with great heterogeneity in patients with MS and were initially thought to represent a biomarker of demyelination (8, 9). The development of CBAs that recognize the native MOG conformation allowed to define the distinct phenotype of this condition, in particular when a high titer cut-off value is used (10–13). These advances have led to the development of specific diagnostic criteria that required both the presence of a compatible clinical phenotype including myelitis, optic neuritis, acute disseminated encephalomyelitis (ADEM) or brainstem syndromes and MOG-IgG positivity tested through a conformational assay (14, 15). The accumulating evidence of differences in clinical-MRI features, relapse risk, treatment, and outcome led to the concept that patients with MOG antibodies are affected by a distinct syndrome that differs from MS and AQP4-IgG-seropositive NMOSD. The term MOG antibody-associated disease (MOGAD) was thus coined to characterize these patients with autoimmune oligodendrocytopathy (16).

**Figure 1:**
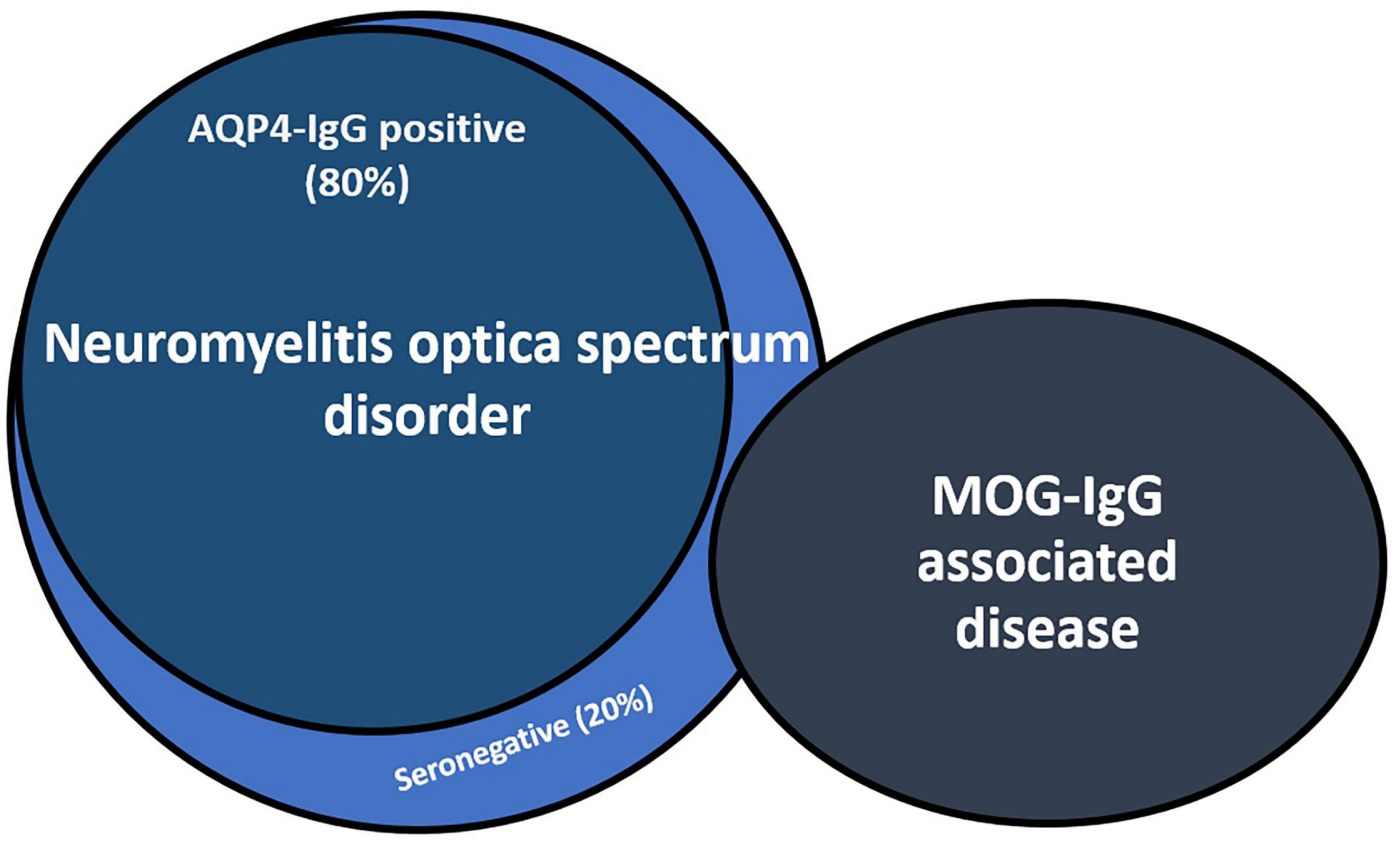
Relationship between aquaporin-4-IgG seropositive and seronegative neuromyelitis optica spectrum disorder (NMOSD) and myelin oligodendrocyte glycoprotein antibody-associated disease (MOGAD). About 30% of NMOSD seronegative patients results positive for MOG-IgG. AQP4-IgG, aquaporin-4 antibodies; MOG-IgG, myelin oligodendrocyte glycoprotein antibodies.

Despite advances in MOG and AQP4 antibody testing, up to 30% of patients with NMOSD remain seronegative for these antibodies. Seronegative NMOSD remains an elusive condition that poses relevant challenges to clinicians in terms of diagnosis and treatment because of its variable prognosis and outcomes (Figure 1).

Over the last few years, different studies have focused on the clinical and paraclinical characterization of NMOSD and MOGAD as distinct demyelinating disorders, but, despite the recent advances, many questions still remain unanswered. The disease course of these conditions, particularly of seronegative NMOSD and MOGAD patients, is unpredictable, with 50% of patients having a monophasic course. There are currently no clinical, paraclinical, or radiological markers that can predict a monophasic or relapsing disease course, which could require different therapeutic choices. In a similar yet different perspective, although the relapsing course of AQP4-IgG seropositive NMOSD always requires immunosuppressive drugs, there are no clinical predictors of treatment response.

In this uncertain setting, the identification of reproducible, repeatable, and easily accessible biomarkers could be of utmost relevance to guide clinicians in these diagnostic and therapeutical challenges.

## Diagnostic Criteria and Assays for Diagnosing NMOSD and MOGAD

### Neuromyelitis Optica Spectrum Disorder

Based on the most recent 2015 NMOSD diagnostic criteria, the diagnosis of AQP4-IgG NMOSD requires the presence of (i) 1 clinical core feature including optic neuritis, acute transverse myelitis, APS, acute brainstem syndrome, narcolepsy or acute diencephalic lesion or symptomatic cerebral syndrome with typical NMOSD brain lesions, (ii) positive testing for AQP4-IgG (CBAs are recommended), and (iii) the exclusion of alternative diagnoses such as MS, sarcoidosis, infections, neoplasms, and paraneoplastic disorders. The diagnosis of seronegative NMOSD relies on the presence of at least two different clinical manifestations of NMOSD, one being ON, transverse myelitis or AP, with evidence of consistent demyelinating lesions on MRI and negativity of AQP4-IgG tested with the best available assay (6). CSF analysis usually demonstrates pleocytosis (observed in up to 51% of cases), whereas CSF restricted oligoclonal bands are found in only about 16% of patients (17).

AQP4-IgG can be detected using different laboratory techniques such as live- or fixed-CBAs revealed using immunofluorescence or flow-cytometry/fluorescence-activated-cell-sorting (FACS), ELISA or tissue-based assays. These assays, with the exception of tissue-based assays, recognize one of the two isoforms of AQP4, i.e., M1 or M23. The presence of antibodies against each specific isoform has not been associated with different clinical features or outcomes (18). The comparison between diagnostic assays have proved that CBAs, either live or fixed, are the most accurate test for the detection of AQP4-IgG (accuracy: 99.3%) and that M23 expressing cells may perform better than M1-based assays (4, 19). Therefore, live CBAs which use the M1 isoform are the most accurate diagnostic test.

Since the detection of AQP4-IgG is fundamental for treatment decisions, assays such as tissue-based assays and ELISA, whose sensitivity ranges between 60 and 78% and may lead to false negative results (20), are not preferred. Indeed, some patients that tested positive on ELISA but negative with CBA had alternative diagnoses identified suggesting a potential for false positivity (21). Even though early reports preferred immunofluorescence over FACS (19), live cell-based assays (either live or fixed) either detected by FACS or by visual immunofluorescence have very high specificity (22). Immunohistochemistry may be useful to detect the typical AQP4-IgG staining patterns on rodent tissue composite, but then AQP4-IgG presence should be confirmed using CBAs (23).

Finally, CSF testing for AQP4-IgG is not routinely recommended since it is less sensitive than serum testing (24). Indeed, almost all CSF positive patients are positive in serum at high titers (24).

### Myelin-Oligodendrocyte Glycoprotein Antibody-Associated Disease

The diagnosis of MOGAD relies on MOG-IgG detection by CBA in patients with compatible clinical-MRI phenotypes, including ADEM or encephalitis, brainstem syndromes, transverse myelitis (often longitudinally extensive with central gray and conus involvement), and ON (typically longitudinally extensive with >50% of the optic nerve length affected often accompanied by perioptic gadolinium enhancement on MRI). The ON attacks are usually associated with optic disc edema and can be bilateral, recurrent, and show steroid dependence. Pleocytosis is found in 38–55% of patients while CSF restricted oligoclonal bands are rarely detected in this condition (6–12% of patients) (25–28).

Testing for MOG-IgG in serum through CBAs, both with FACS or visual based indirect immunofluorescence, using full-length human MOG and Fc or IgG1 as secondary antibodies is recommended, although CSF testing may help in cases of patients with a negative serum test and phenotype suggestive of MOGAD (14, 15, 29).

The diagnosis of MOGAD strongly relies on MOG-IgG detection so that the accuracy of diagnostic assays is of utmost importance. According to a multicenter international comparative study (30), live-CBAs (with either FACS or immunofluorescence detection) have the greatest concordance (96%), while fixed-CBAs show a slightly lower value (90%) for the diagnosis of MOGAD. ELISA showed no concordance and was unable to distinguish positive and negative patients and thus this method should not be used for diagnostic purposes of MOGAD. Unfortunately, concordance was overall low even for CBAs for sera with borderline/low positivity, which highlights the importance of testing MOG-IgG only when the pre-test probability is high, in order to avoid false-positive results (31). Although diagnostic criteria do not recommend testing MOG-IgG in CSF, some recent studies have reported some patients with MOG-IgG positivity only in the CSF (not serum) who had a clinical and neuropathological MOGAD phenotype (29, 32). Therefore, seronegative patients whose clinical phenotypes are strongly suspicious for MOGAD may benefit from MOG-IgG testing in the CSF, because up to 4–7% of these patients may harbor CSF-restricted antibodies. Patients with CSF restricted MOG-IgG have similar clinical phenotype in comparison with seropositive ones, with the notable exception of isolated optic neuritis, which is uncommon in patients with CSF-restricted MOG-IgG (29, 33, 34). Finally, MOG isoforms (7) and IgG subclasses (35) binding analysis have been performed, but they are currently used for research purposes only.

## Potential Biomarkers of NMOSD and MOGAD

The role of biomarkers in NMOSD and MOGAD is vital to 1. Aid clinicians in differentiating these conditions from typical MS; 2. Determine the relapse risk; 3. Define disease prognosis; 4. Assist treatment choices.

**Figure 2:**
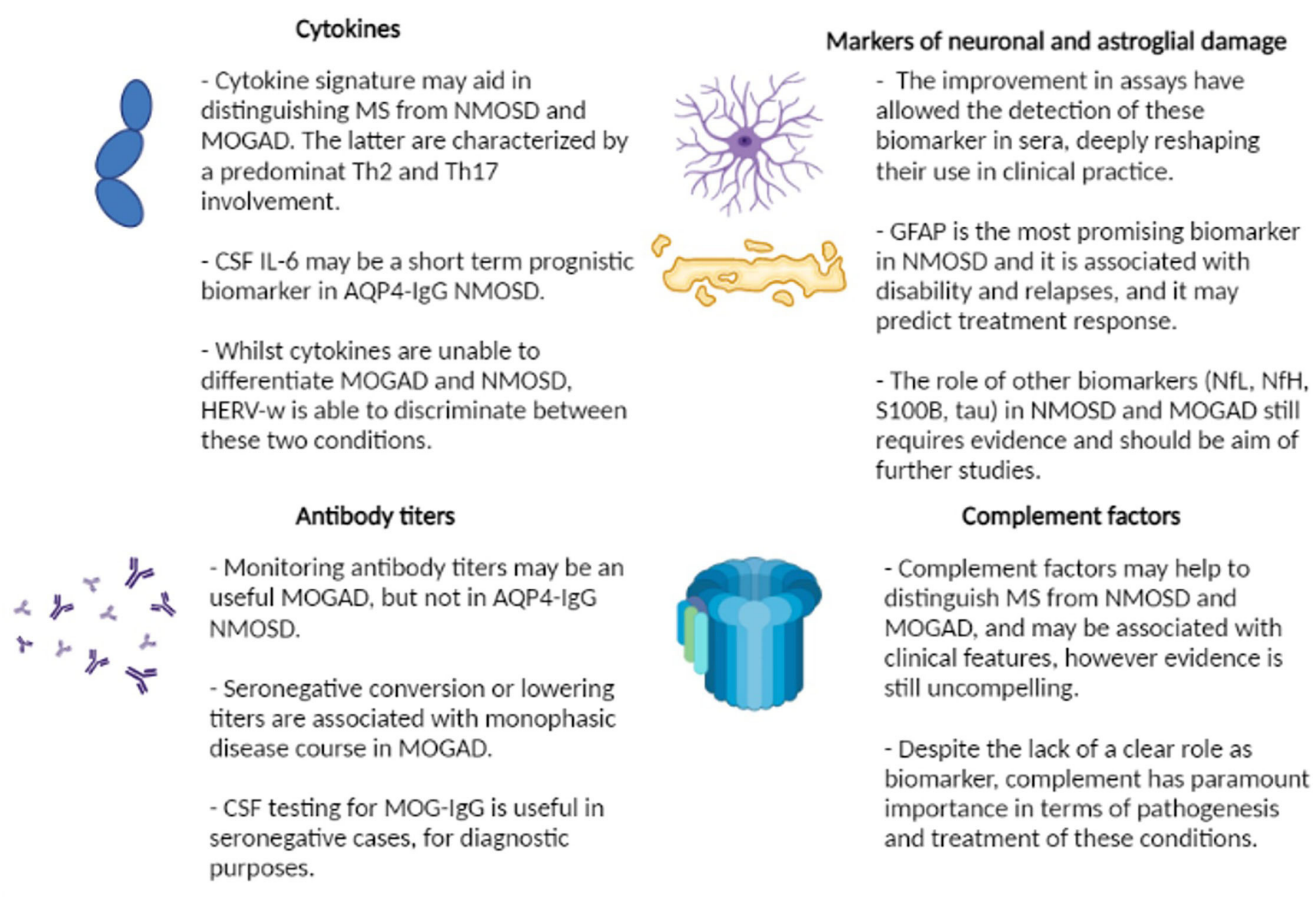
Summary of current evidence regarding the role of biomarkers in neuromyelitis optica spectrum disorder (NMOSD) and myelin oligodendrocyte glycoprotein associated disorder (MOGAD). MS, multiple sclerosis; Th, T-helper; CSF, cerebrospinal fluid; IL-6, interleukin-6; AQP4-IgG, aquaporin4 antibodies; HERV-w, human endogenous retroviruses; MOG-IgG, myelin oligodendrocyte glycoprotein antibodies; GFAP, glial fibrillary acid protein; NfL, neurofilament light chain; NfH, neurofilament heavy chain.

Current biomarkers are related to different pathogenetic aspects of the diseases and may be broadly classified into these 4 groups (Figure 2):

- Antibody titers and persistence
- Complement proteins
- Cytokines and other immunological markers
- Markers of neuronal and astroglial damage.

### Antibody Titers and Persistence

Antibody titers reflect antibody concentration and thus may represent a useful biomarker for CNS disorders associated with pathogenic antibodies. Indeed, in other conditions such as NMDA-R encephalitis, higher titers at diagnosis predict a worst outcome and increases in titer in the CSF are associated with relapses (36). MOG-IgG titers have also an important diagnostic role because only high titers have been consistently associated with a defined phenotype, whilst low positive titers me be found in other neurological conditions and they be also found in atypical phenotypes which are not related to MOGAD (31, 37).

However, the role of antibody titers in patients with AQP4-IgG-NMOSD and MOGAD as a prognostic and predictive factor is still debated and differences exist in the potential role of these titers as biomarkers, reflecting the heterogeneity of the underlying biology.

Regarding AQP4-IgG-NMOSD, AQP4-IgG serum titer at the time of attack has been found to be higher in patients presenting with ON (38) and to be associated with the severity and outcome of the event (blindness, length of myelitis on MRI) (39, 40), however these findings were not consistently replicated in other studies (41, 42) and therefore the utility of AQP4-IgG titers in predicting disease is unclear. Furthermore, antibody titers may fluctuate during disease course, particularly during the relapse and remission phase. Several studies demonstrated that patients have higher AQP4-IgG titers at the time of relapse when compared to the remission phase (39, 43–45) however this difference was not seen in all studies (42, 46). An increase of antibody titers may precede relapses, but it is important to note that some patients with high or increasing titers may not experience relapses (43–45, 47). Similarly, patients with low or stable titers, initially thought to be predictive of monophasic disease (39, 48), may also experience relapses (43, 45, 46). Intriguingly, up to 55% of patients may become seronegative during their disease course and experience subsequent relapses with a concomitant increase of AQP4-IgG titers (45), limiting the prognostic role of seronegative conversion after onset.

AQP4-IgG titer in the CSF may have a different dynamic when compared to serum titer. CSF levels of AQP4-IgG are mainly related to the antibody leak in the CNS due to an increased permeability of the blood-brain barrier (49). AQP4-IgG in the CSF may be more frequently detected during attacks (85%) rather than during remission (49) and their ratio to serum titers is also increased in the acute stage (50). CSF titers may decrease after a relapse, whilst serum titers remain stable, and their reduction has been reported to be associated with clinical improvement (51). However, there are less studies on the prognostic utility of AQP4-IgG in the CSF because the difficulty in obtaining CSF and the observation that serum is more sensitive than CSF as a biomarker of NMOSD.

Finally, some studies have found that antibody titers may decrease after immunotherapy (39, 43) and may increase when treatment is suspended (43). The reduction of antibody titers after treatment has been reported to help predict responders to rituximab (44). However, these findings have not been consistently replicated across different studies (41, 46), which limits the use of AQP4-IgG as a clinical biomarker to monitor treatment efficacy.

MOG-IgG titers represent a complex biomarker, influenced by several factors including age and clinical presentation at onset. Studies have suggested that MOG-IgG titers are higher during relapses (13, 26) and may be highest in patients presenting with myelitis (12) or ADEM (10, 52). A study by Cobo-Calvo et al. found that antibody titers at the first episode was related with acute disability at onset, but not with long-term outcome or a predictor of relapse (53). In contrast, a study by Hennes et al. found a high titer at onset predicted a relapsing course in a pediatric cohort (54), and therefore usefulness of the initial MOG-IgG titer level in prognostication remains unclear.

MOG-IgG titer often declines over time after the first demyelinating attack. In pediatric patients with ADEM, MOG-IgG titers are usually high at onset, and may subsequently decline or become seronegative, in about 50% of patients regardless of the onset titer (52, 55). This may have important clinical relevance because seronegative pediatric patients have been shown to have a significantly lower risk of relapse (55, 56). The persistence of high MOG-IgG titers may predict relapses in this setting (54), but up to 72% of persistently positive pediatric ADEM patients will also remain monophasic. When including both adult and pediatric ADEM patients, Lopez et al. found that only 12% of patients that became seronegative experience relapses, compared to 88% of persistently seropositive ones (15). Several other studies, including both pediatric and adult MOGAD patients with a variety of phenotypes, have found that seronegative conversion is associated with a lower relapse risk in MOGAD patients (25, 26, 35, 57). While 50% of pediatric MOGAD patients become seronegative, only roughly 25% of adults become seronegative. It should be noted that while persistent seropositivity may be associated with an increased risk of relapse, not all patients with persistent seropositivity will inevitably experience relapses. Indeed, Juryncyzk et al. found that among 72% of patients that remained persistently seropositive after the first attack, only 59% experienced further relapses (25), thus proving that persistently MOG-IgG seropositivity is not always associated with further clinical events. In addition, some patients that become seronegative can rarely relapse and become seropositive at the time of the relapse (55).

The role of antibody titers in AQP4-IgG-NMOSD and MOGAD is extremely complex and likely differs in the two conditions. The predicting role of AQP4-IgG titers regarding outcomes, disease course or relapses is vastly controversial due to the inconsistency of the results, which may potentially be explained by the different methods used to determine the titer of AQP4-IgG. Some studies used CBAs (39), while others used fluorescence-based immunoprecipitation assay (FIPA) (49) or ELISA (45). Beside methodological differences, it is clear that some relapses in patients with AQP4-IgG-NMOSD occur regardless of AQP4-IgG titer at onset and its fluctuation during the disease course does not always correlate with disease activity. These features make AQP4-IgG titer a non-optimal biomarker for this condition.

On the contrary, in patients with MOGAD the dynamics of MOG-IgG titers may be helpful in identifying patients that will experience a monophasic disease course since patients that become seronegative will more frequently be monophasic. On the other hand, persistently positive MOG-IgG patients may have a higher chance of relapse, but can also have a monophasic disease course, and therefore, in this setting, antibody titers are less helpful as predictors. Of note, the results found in patients with MOGAD may be more consistent because all recent studies use MOG-IgG testing with conformational CBAs.

### Complement Proteins

Both AQP4-IgG and MOG-IgG predominantly belong to the IgG1 subclass and thus may activate the complement cascade. The role of complement activation was noticed even before the discovery of AQP4-IgG (58) and it is now clearly demonstrated (59).

Complement activation and complement-associated cell killing (60) have relevant therapeutic implications in AQP4-IgG NMOSD, including the successful randomized clinical trial of eculizumab in prevention of relapses (61).

On the other hand, the role complement in patients with MOGAD is still a matter of debate (62, 63).

Complement proteins are a potential biomarker of AQP4-IgG NMOSD and have been proposed to be useful to distinguish NMOSD from other demyelinating disorders, such as MS. Indeed, many studies used patients with MS as controls along with patients without inflammatory neurological disorders. The comparison of complement proteins between these groups have regrettably led to inconsistent results. Indeed, an initial study found that C4d and sC5-C9 complex were higher in NMOSD patients in comparison with healthy controls and MS patients (64). Similar results regarding the increase of sC5b-9 complex in serum and CSF were replicated in other studies (65, 66), which failed to give consistent results regarding C3 and C4 proteins. C3 was found to be equal (66, 67) or increased when compared to healthy controls (65). Similarly, C4 was reported to be increased (64) and decreased (65) in NMOSD patients. Kuroda et al. found that CSF concentrations of C3 and C4 did not segregate in the different groups (67). Hoellou et al. found that CSF C3 and C4 were similar in pediatric MS and MOGAD, but C5a was higher in the latter group (68).

More recent studies have systemically assessed complement proteins, activation products and regulators in patients with NMOSD in serum (69) and CSF (70). These studies highlighted that plasmatic C1Inh, C5 may be helpful to distinguish NMOSD vs. healthy controls, whilst C1Inh and C5b–C9 could segregate MS and NMOSD (69). Finally, CSF levels of C3, C4, C5, C9, FH, FHR, and C1Inh may be used to differentiate the two inflammatory conditions (70).

Some studies have reported an association between complement levels and disease activity in patients with AQP4-IgG NMOSD. In particular, serum C3 levels were found to correlate with EDSS (65) and CSF C5a values were associated with MRI activity and delta EDSS (67). In addition, a study by Veszeli et al. suggests complement may be altered, although not activated, even in patients in remission and under disease-modifying treatment (71).

Recent studies have tried to distinguish specific complement signatures in AQP4-IgG NMOSD compared to MOGAD patients. Patients with AQP4-IgG NMOSD had lower (72) or comparable (73) levels of C3 and lower concentration of C4 when compared to patients with MOGAD (72, 73). According to a different study by Hakobayn et al. Bb, C4a and C5a components were higher in AQP4-IgG NMOSD patients, while iC3b and C5b–C9 were higher in MOGAD patients (69). On the contrary, Keller and colleagues surprisingly found that all complement levels with the exception of C3a were higher in patients with MOGAD when compared to NMOSD (63). These discordant findings may be related to the fact that samples were obtained during remission (73) or at first event (72). This study also found that patients with MOGAD have higher complement levels when compared to healthy controls and MS (63). However, the complement levels did not differ between patients with a monophasic or relapsing disease course and did not differ between patients during relapse or in remission (63). Therefore, its role as a biomarker of disease is limited. Future studies are required to confirm complement's role in MOGAD (74).

Overall, complement may be a useful biomarker to discriminate patients with MS, MOGAD, and NMOSD, but studies have been mixed and larger prospective studies are required. Some studies have also demonstrated that complement may be associated with some clinical and radiological indexes of disease activity and severity. However, results among different studies have led to discordant findings, probably due to methodological and sampling issues (e.g., use of CSF or serum, sampling during remission or active disease). Despite these inconsistencies, these studies show that complement may play a key role in the pathogenesis of these disorders and may represent a potential therapeutic target.

### Cytokines and Other Immunological Biomarkers

The different pathogenesis between MS and other antibody-mediated demyelinating disorders has led to the question whether cytokine signatures could differ in these conditions, and thus whether cytokines could represent a useful tool in the differential diagnosis.

The importance of studies on cytokines, and in particular interleukin-6 (IL-6), underlies the success of the recent randomized clinical trial on satralizumab, which was found to be more effective in AQP4-IgG NMOSD, but less effective for seronegative NMOSD (75). Similarly, tocilizumab was found to reduce the risk of relapses in both in AQP4-IgG NMOSD and MOGAD (76, 77).

Early studies on “opticospinal multiple sclerosis” (OSMS), which likely were cases of NMOSD, have demonstrated that a discrete number of cytokines, in particular T-helper 2 (Th2) and T-helper 17 (Th17) related ones, could differentiate OSMS from both unaffected patients and patients with other forms of acute myelitis (78) or MS (79).

Among these cytokines, CSF levels of Th2-related IL-6 seem to have a prominent role in patients with NMOSD. Indeed, IL-6 is increased during relapses (80) and it may be a useful biomarker to discriminate between MS and NMOSD (81–85). IL-6 has been shown to correlate with the length of myelitis (83), EDSS (82), particularly in untreated patients (86), and correlate with markers of glial and myelin damage [as glial fibrillary acid protein (GFAP) (84, 86, 87) and myelin basic protein (MBP) (87)]. CSF IL-6 levels may also predict the outcome after a relapse and the occurrence of further short-term relapses (88). Of note, AQP4-IgG seropositive and seronegative NMOSD patients have different concentrations of IL-6 (80, 84), with seropositive patients harboring the highest concentration. In contrast to CSF testing, several studies have shown that measuring IL-6 in serum/plasma is not useful in discriminating between NMOSD and other conditions (84, 85, 89). However, Monteiro et al. (90) demonstrated that plasma concentrations of IL-6 and interleukin-17 (IL-17) are associated with relapses and disability measured with the EDSS score in patients with NMOSD.

Other relevant cytokines analyzed in NMOSD include the Th17 related-ones -IL-8 (85, 86) and IL-17 (82, 86, 89, 90)- and the T-helper regulatory interleukin-10 (IL-10) (84, 87). Individual reports have also highlighted that other chemokines or related molecules, such as CXCL8, CXCL10 (82), CXCL13 (91), BAFF, and APRIL (92), could be useful biomarkers in NMOSD. However, they have been analyzed in only few studies and further evidence is required.

As for MOGAD, studies on cytokines have demonstrated that patients with MOGAD share a similar cytokine signature to that observed in patients with NMOSD, with a predominant involvement of IL-6, IL-8, IL-10, and IL-17 (68, 85, 87, 93) that may be used to distinguish MOGAD from MS but not from NMOSD. Intriguingly, human endogenous retroviruses (HERVs), a novel biomarker that has been studied mainly in MS (94), has shown promising results in terms of differentiating among demyelinating diseases. HERV-w peptides have been found in 78% of patients with MS and in 8% of patients with NMOSD, regardless of AQP4-IgG serostatus (95), suggesting a potential role in differentiating these entities. Moreover, a recent study by Arru et al. found HERV-w peptides in 91% of patients with MOGAD compared to only 32% in AQP4-IgG NMOSD, which suggests it may be a helpful diagnostic biomarker to distinguish between these two conditions (96).

Taken together, the role of cytokines and immunological markers may be useful in NMOSD and MOGAD to predict the short-term outcome and to identify patients that will experience a new attack. However, the role of cytokines, and in particular of IL-6 which has the most solid evidence as a biomarker, is limited by the fact that most of the clinical and prognostic correlations are found with CSF rather than serum IL-6 levels. This limitation is relevant because it prevents monitoring cytokines levels over time and the use of these biomarkers in longitudinal studies.

On the other hand, cytokines levels may be useful to distinguish NMOSD from MS, which has relevant clinical and therapeutical implications. MOGAD and NMOSD share similar cytokines signature and therefore cytokines may not be useful in the differential diagnosis. However, HERV-w peptides have shown promising results in differentiating the two conditions.

### Markers of Neuronal and Astroglial Damage

Markers of neuronal and astroglial damage represent a broad spectrum of molecules that are released in the CSF after CNS injury. The two main molecules that have been the most studied within this category are neurofilament light chain (NfL) and GFAP, although other molecules such as astrocytic marker S100B, MBP, neurofilament heavy chain (NfH), and tau proteins have also been analyzed in a few studies in patients with NMOSD and MOGAD.

Briefly, neurofilaments are intracellular proteins involved in radial growth and stability of axons that are released, together with other axonal cytoskeletal proteins, into the CSF after neuroaxonal damage. NfL has been demonstrated to be a promising biomarker (97), useful in different neurological conditions such as MS, dementia, stroke, traumatic brain injury, Parkinson's disease, Huntington disease, encephalitis, peripheral neuropathies, and amyotrophic lateral sclerosis (98–102). On the other hand, GFAP represents the main cytoskeletal protein of mature astrocytes, and it is also involved in regeneration, plasticity, and reactive gliosis (103). GFAP is a promising biomarker in the setting of traumatic brain injury, MS, frontotemporal dementia, and other diseases (104, 105).

Both NfL and GFAP are released in the CSF after axonal or astroglial injury, respectively, and a small proportion of these proteins can also be detected, at lower concentration, in the blood. First- and second-generation assays such as ELISA or immunoblot could detect these biomarkers properly only in the CSF because of the lack of sensitivity. New generation assays (such as electrochemiluminescence and, particularly, single-molecule array -SIMOA-) are able to detect these molecules in the blood with high sensitivity, thus allowing the conduction of longitudinal studies and monitoring of their values over time (99).

Initial studies on patients with NMOSD were performed with ELISA on CSF samples and were thus limited by the lack of longitudinal follow-up or remission phase data, given the difficulties in repeating lumbar punctures outside the setting of an acute event. There are only two studies using ELISA in serum, which provided conflicting results. In particular, Storoni et al. found higher concentrations of serum GFAP in patients with AQP4-IgG related ON (106), while this was not found by Fuji and colleagues (107). Most of these earlier studies, predominantly performed on CSF, showed that the makers of astrocytic damage, GFAP and S100B, were higher in patients with NMOSD when compared to patients with MS or healthy controls (84, 87, 107–112). One recent study that combined GFAP and glutamine synthetase (GS) analyses found that markers of astrocytic damage were higher in patients with AQP4-IgG NMOSD when compared to MS, but also found some seronegative NMOSD patients with high levels of GFAP and GS (113). Other studies have demonstrated that the values of GFAP and S100B are lower in seronegative patients when compared to AQP4-IgG positive NMOSD patients (87). Given the ability of GFAP to discriminate between NMOSD and MS (112), the increase of GFAP values was proposed as a supportive criterion for NMOSD diagnosis (110). However, the utility of GFAP and S100B in discriminating among MOGAD, AQP4-IgG NMOSD, and double seronegative patients was not consistent according to the few studies which tried to address this issue (111, 112).

Regarding the associations with clinical features, GFAP levels in the CSF have been found to be related to EDSS on relapse (108, 109), EDSS during remission (112), EDSS at 6 months follow-up (109), and the number of spinal cord segments involved (108, 109). S100B values have been associated with the number of spinal cord segments involved (108, 109) and EDSS on relapse (109, 112) and on remission (112). GFAP have been shown to be more elevated in patients with myelitis when compared to those with brain lesions and ON (109, 112) and reduces after treatment (108, 109).

Evidence regarding other biomarkers tested with ELISA on CSF is more limited. MBP was found to be higher in both MOGAD and AQP4-IgG NMOSD patients when compared to MS (87, 111), although this association was not consistent with a previous study (109) showing MBP correlation with EDSS and length of myelitis. NfH values were found to be higher in NMOSD when compared to controls patients (110, 114), but similar to those detected in patients with MS (115). On the other hand, NfL levels were higher in NMOSD in comparison to both healthy controls and patients with MS (115). NfL values correlated with disability in both NMOSD and MS, whilst NfH was associated with disability in MS only. Despite the lack of association with disability, the increase of NfH concentration persisted after relapses in NMOSD (114). Intriguingly, one study analyzing NfL concentration in seronegative NMOSD patients failed to detect differences in comparison with non-inflammatory controls, in contrast to results found in AQP4-IgG NMOSD patients (84).

The advent of next-generation assays, particularly SIMOA, have reshaped the analysis of these biomarkers by allowing a more consistent and reproducible measure in serum, which has the clear advantage of being more accessible than CSF. Recent studies focused on the role of serum GFAP (sGFAP) and NfL (sNfL) during the relapse and remission phase of AQP4-IgG-NMOSD and MOGAD, their association with clinical and paraclinical variables and their role in predicting further relapses.

Most of studies have found higher levels of sGFAP and sNfL in patients with NMOSD and MOGAD compared to healthy controls (116–121), although one study which analyzed samples obtained in sustained clinical remission failed to confirm this finding (122). Intriguingly the few studies which included seronegative NMOSD patients did not find differences of sNfL concentration between seronegative NMOSD patients and healthy controls (116), thus suggesting a different underlying biology.

The role of biomarkers of tissue damage in differentiating MS and other demyelinating disorders is still a matter of debate and data are likely influenced by the timing of sampling (relapse vs. remission). For example, Mariotto et al. found higher concentrations of sNfL in patients with MOGAD and NMOSD when compared to MS in samples obtained mostly during disease activity (116), while Watanabe et al. did not find a difference in sNfL values between MS and NMOSD when samples were obtained during remission (118). Other studies found discordant results with similar levels of sNfL between the two conditions (123) or even higher values in patients with MS (124). On the other hand, most studies have found that sGFAP concentration is higher in NMOSD (118, 124).

As for the difference between patients with NMOSD and MOGAD, one study found that GFAP levels are higher in patients with NMOSD (125), whilst tau and sNfL were comparable. However, other reports did not find any differences between the two conditions in terms of sNfL levels (116, 124) or sGFAP (124). Samples obtained during relapses may help to differentiate between MOGAD and NMOSD. For example, Kim et al. found that tau protein was increased during MOGAD relapses, while sGFAP and sNfL levels were increased during AQP4-IgG NMOSD relapses (125). In addition, the relationship between sGFAP and sNfL (i.e., the sGFAP/sNfL ratio) may be a more specific index of astrocytic damage and may be able to better differentiate these conditions (118, 119, 124) but future larger studies are required to confirm these findings.

Regarding prognosis, sGFAP plays a key role in patients with AQP4-IgG NMOSD and the dynamics of this molecule has been elucidated by a prospective study conducted during the NMO-Momentum trial for inebiliziumab (126). Higher sGFAP levels were found in patients with AQP4-IgG NMOSD, which was also related with age and EDSS. The NMO-Momentum trial found that higher baseline levels of sGFAP was associated with a 3-fold increased risk of subsequent relapses, and within 1 week from a relapse, sGFAP concentration increased and started to decline 5 weeks after the event. The severity of relapse was correlated with the sGFAP concentration. However, it was noted that patients may experience an increase of sGFAP without concomitant relapses, though in these cases minor neurological symptoms (not defined as relapses) or asymptomatic MRI lesions may be detected. The NMO-Momentum trial also showed that treatment with inebilizumab reduced the concentration of sGFAP in treated patients and reduced the risk of relapse. Intriguingly, patients may experience relapses during treatment with inebilizumab, however sGFAP concentrations do not significantly increase, suggesting that relapses on treatment may induce less astrocytic damage or may be a reflection of inebilizumab stabilizing the blood-brain barrier (126).

Other studies in AQP4-IgG NMOSD have also demonstrated that sGFAP is higher during relapses (118, 119, 121, 124, 125), that higher concentration of sGFAP may predict the occurrence of relapses (118, 122), and that sGFAP concentration may discriminate between stable and active disease (119, 121, 125). sGFAP concentrations decrease over time after a relapse (119) but may remain elevated during the remission stage (118, 121) and then normalize after 3–4 months (122, 127). Of note, sGFAP levels may subtly increase during inter-attack periods (127) or may increase over time in absence of immunotherapy (119), suggesting possible subclinical ongoing astrocytic damage in AQP4-IgG NMOSD.

Regarding the role of sNfL in AQP4-IgG NMOSD, most studies have found that serum sNfL to be increased during relapses (121, 125) and to slowly decrease over time (119, 127) or normalize after treatment (118, 121), reaching comparable levels to that observed in healthy controls in sustained remission (122). However, one single study detected stable sNfL values during relapses (124).

Finally, serum sGFAP values have been associated with EDSS score (118, 121–125), Multiple Sclerosis Composite Scale (122), the occurrence of myelitis (118, 124), and a recent relapse (118, 121). Conversely, serum sNfL have been associated with EDSS only (118, 121, 123, 124).

The role of these biomarkers in patients with MOGAD is less defined. sNfL and sGFAP may increase during relapses (124) and the increase of sNfL is more marked in severe attacks (117). In patients with multiple relapses, sNfL have been found to be increased only during the first relapse and then remain stable, supporting the role of the first attack in determining long-term disability (128). On the contrary, one aforementioned study has demonstrated that only serum tau, and not sGFAP or sNfL, increases during relapses (119). Another study found that sNfL increases during relapses among patients with MOGAD, but sGFAP did not increase (129). As in NMOSD, sNfL levels reduce over time after an acute attack (128) and during remission they may be similar to controls (122, 129).

In MOGAD, sNfL values are associated with EDSS (117, 124, 125), are higher in pediatric patients presenting with encephalopathy (130) and both sGFAP and sNfL have been found to be associated with a recent brain lesion (124). In addition, tau concentration has been found to be associated with EDSS (125). Serum biomarkers may also have a diagnostic role when associated with neuroradiological findings: indeed, the ratio between sNfL and the T2 lesion area on MRI (neurofilament light chain/area ratio) may discriminate between spinal cord infarction and other acute myelopathies, such as NMOSD or MOGAD (131).

Overall, current evidence shows that molecules related to neuronal and astroglial damage are promising biomarkers in NMOSD, and particularly GFAP seems to be a reliable marker of disease activity. The improvements in the diagnostic assays have allowed to study the dynamics of these molecules in the serum during and after relapses, and even in remission. The presence of baseline elevated concentrations of GFAP predicts the occurrence of relapses in NMOSD and thus it may be used to identify patients that may require more aggressive treatment. Similarly, GFAP could be used as a marker of treatment response to promptly identify non-responders. On the other hand, the increase of GFAP before a relapse may be useful to monitor patients without therapy and to promptly treat them when increasing concentrations of this biomarker are detected. Finally, GFAP may be useful to determine subclinical progression in patients with NMOSD. Despite these promising studies, the role of GFAP in clinical practice has not been established and reliable cut-offs are not yet available to determine remission and relapses. Although some studies tried to determine clear cut-offs for disease status, the included populations were small and methodological issues may hinder the reproducibility of these values.

The role of these biomarkers in patients with MOGAD is still uncertain and requires more clinical evidence, although tau and NfL may be promising molecules, given their association with disability, even though their association with relapses is still unclear and deserves further study. Finally, GFAP and NfL may be useful markers to differentiate MS from MOGAD and NMOSD, although studies have been mixed. The adoption of the GFAP/NfL ratio, which represent a marker of astrocytic damage that accounts for both NfL and GFAP, may better discriminate among different demyelinating disorders, but needs to be explored in future studies.

## Conclusions

Ideal biomarkers should be precise, easily accessible, reproducible, and most importantly be able to predict the disease course and aid in the differential diagnosis. Biomarkers are an emerging and promising field that may help clinicians in the management of patients with MOGAD and NMOSD, and their incorporation as surrogate endpoints in clinical trials is warranted.

Regarding the application of biomarkers in the clinical practice, this review has shown that (a) seronegative conversion of MOG-IgG may be helpful in MOGAD to distinguish monophasic and relapsing patients, so that monitoring MOG-IgG titer over time is recommended. On the contrary, evidence does not support monitoring in AQP4-IgG in NMOSD. Even if CSF testing of AQP4-IgG and MOG-IgG is not routinely indicated, there is growing evidence that evaluating for MOG-IgG in the CSF may be useful to identify rare patients with MOGAD when serum is unrevealing; (b) complement proteins shed light on the pathogenesis of antibody-mediated demyelinating disorders and may support the use of complement-directed therapies, but their role as biomarkers has yet to be defined; (c) cytokines, and in particular IL-6, may be useful to distinguish NMOSD/MOGAD from MS, and may be useful as a short-term prognostic factor; (d) given the advances in assay sensitivity allowing evaluation of proteins released after astroglial or neuronal damage in the serum, these markers of injury are becoming promising biomarkers in these conditions. In particular, serum levels of GFAP have a strong association with AQP4-IgG NMOSD disease course, whilst the contrasting data related to MOGAD and seronegative NMOSD patients warrants additional future studies.

## Author Contributions

AD wrote the first draft of the manuscript. SM and JC performed the first revision of the manuscript including critical additional data. All the authors performed additional revision to the manuscript and gave critical intellectual content. All authors contributed to the article and approved the submitted version.

## Funding

This study was supported by personal research funds of the SM.

## Conflict of Interest

JC is a consultant to UCB and Roche. The remaining authors declare that the research was conducted in the absence of any commercial or financial relationships that could be construed as a potential conflict of interest.

## Publisher's Note

All claims expressed in this article are solely those of the authors and do not necessarily represent those of their affiliated organizations, or those of the publisher, the editors and the reviewers. Any product that may be evaluated in this article, or claim that may be made by its manufacturer, is not guaranteed or endorsed by the publisher.
